# Case report of a modified Meso-Rex bypass as a treatment technique for late-onset portal vein cavernous transformation with portal hypertension after adult deceased-donor liver transplantation

**DOI:** 10.1097/MD.0000000000007208

**Published:** 2017-06-23

**Authors:** Dongdong Han, Rui Tang, Liang Wang, Ang Li, Xin Huang, Shan Shen, Jiahong Dong

**Affiliations:** Department of Hepatopancreatobiliary Surgery, Medical Center, Tsinghua University, Beijing Tsinghua Changgung Hospital, Beijing, China.

**Keywords:** liver transplantation, Meso-Rex bypass, portal hypertension, portal vein cavernous transformation, portal vein thrombosis

## Abstract

**Rationale:**

Portal vein thrombosis is a complication after liver transplantation and cavernous transformation of the portal vein (CTPV) is a result of portal vein thrombosis, with symptoms of portal hypertension revealed by an enhanced CT scan. Meso-Rex bypass is an artificial shunt connecting the left portal vein to the superior mesenteric vein and is mainly used for idiopathic cavernomas. This technique is also used for post-transplant portal vein thrombosis in pediatric patients thereby bypassing obstructed sites of the extrahepatic portal vein. Here we report about an adult patient who was treated by connecting the cystic part of the portal vein to the splenic vein instead of the superior mesenteric vein.

**Patients concern:**

An adult male patient with post-liver transplantation portal vein cavernous transformation suffered from hypersplenism and elevated hepatic enzymes.

**Diagnosis:**

The last follow up revealed irregular and obvious hypersplenism, and splenomegaly had occurred, while an enhanced CT scan revealed serious esophagogastric varices and CTPV in addition to occluded right and common PV trunks.

**Intervention:**

The patient was treated by connecting the cystic part of the portal vein to the splenic vein instead of the superior mesenteric vein.

**Outcome:**

After the operation, a satisfactory velocity was confirmed 1 month postoperatively and the shunt still remained patent at the 6-month postoperation follow-up.

**Lessons:**

A Meso-Rex bypass intervention connecting the left portal vein to the splenic vein instead of the superior mesenteric vein after liver transplantation in an adult patient with right and common portal vein occlusions has been successfully performed as an alternative approach.

## 1 Introduction

The sponge-like appearance of multi-hilar collaterals in the cavernous transformation of the portal vein (CTPV), as a result of portal vein thrombosis (PVT), leads to the formation of collateral vessels such as the paracholedochal vein in the hepatocolic and hepatoduodenal ligaments.^[1]^ These collateral shunts generate frequent secondary complications after liver transplantation in pediatric patients.^[2]^ Cavernous transformation may appear 6 to 20 days after the portal vein (PV) obstruction,^[3]^ with concomitant symptoms including hypersplenism, liver dysfunction, and variceal bleeding due to portal hypertension and impaired hepatopetal flow. Meso-Rex bypass (MRB) is a recently established surgical technique^[4,5]^ designed to decrease PV pressure and restore normal blood flow to the liver in post-liver transplantation pediatric patients with CTPV and PVT.^[6]^ In this procedure, portal decompression is achieved by creating a conduit between the mesenteric system via the superior mesenteric vein (SMV) and the left portal vein (LPV), thereby directing mesenteric venous and splenic blood around the obstructed area of the extrahepatic PV and into the liver; commonly an internal jugular vein autograft from the patient is utilized.^[7]^ However, when the SMV is too small, thrombosed, or damaged, other reports have documented alternative methods in which the coronary or splenic veins (SV) are transposed to the intrahepatic PV, in the later instance only after splenectomy.^[8,9]^ This is the first case report about a MRB operation performed in an adult patient connecting the LPV to the SV instead of the SMV after liver transplantation.

## 2 Case report

**Figure 1:**
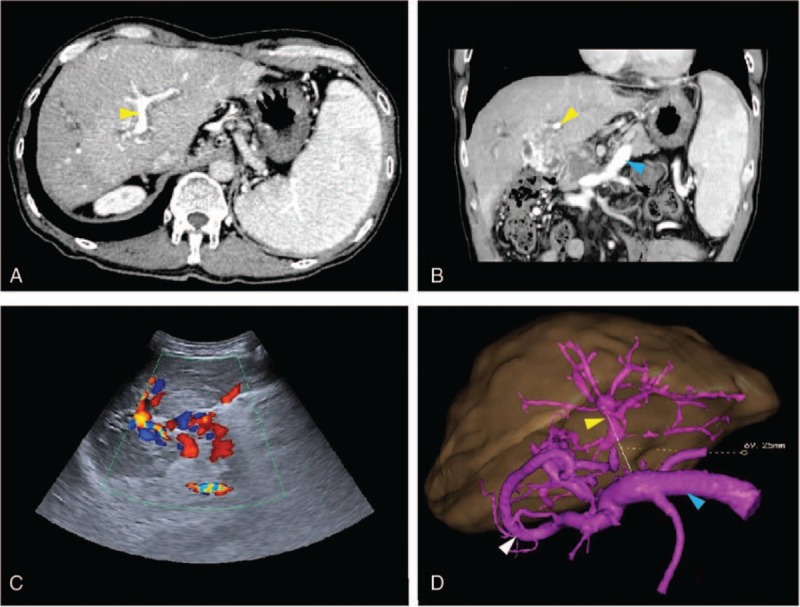
Preoperative enhanced CT scan (A, B) and Doppler US images (C) showed a patent LPV (yellow arrow), SV (blue arrow) and the confluence of the SMV and SV (D). On 3D reconstruction, an obvious varix presented (white arrow). The distance between the LPV and SV, measured to facilitate surgery, was 69.25 mm (yellow line) (D). CT  =  computed tomography, LPV  =  left portal vein, SMV  =  superior mesenteric vein, SV  =  splenic vein, 3D  =  three dimensional, US  =  ultrasound.

A 52-year-old male patient was admitted to our hospital for a post-liver transplantation follow-up; he received liver transplantations twice in another hospital 3 years ago because of fulminant hepatitis B and acute graft failure of the first transplanted liver 1-month postoperation. After the second donor following cardiac death (DCD) whole liver transplantation, the patient recovered well without complications, but blood tests showed decreased leukocytes and platelets with normal liver function during the previous year. His medication included lamivudine and tacrolimus but no anticoagulation or antiplatelet therapy. Follow-ups were every 6 months with an enhanced CT scan once a year. The last follow-up at 3 years post-transplantation revealed irregular and obvious hypersplenism, and splenomegaly had occurred, while an enhanced CT scan revealed serious esophagogastric varices. CTPV was present and the right and common PV trunks were occluded; the left branch, the SV, and the SMV were patent. The diameter of the LPV was 5 mm and Doppler US showed a normal echo of the liver parenchyma, while the PVT reduced the velocity of blood flow to 8 cm/s. Hepatic artery (diameter 4 mm) blood flow increased to compensate, reaching a peak velocity of 73.2 cm/s and a resistance index of 0.73. The diameter of the SV was 1.5 cm. Routine blood examinations revealed leukocytopenia and thrombocytopenia (white blood cells (WBC) 1.34 × 10^9^/L, platelets (PLT) 28 × 10^9^/L, and neutrophils (NEUT) 0.57 × 10^9^/L). Liver function was acceptable: alanine transaminase (ALT) 29.2 U/L, aspartate aminotransferas (AST) 36.4 U/L, alkaline phosphatase (ALP) 111.1 U/L, gamma-glutamyl transferase (GGT) 138.0 U/L, total bilirubin (TBIL) 34.72 μmol/L, and direct bilirubin (DBIL) 18.24 μmol/L. Though the patient suffered no discomfort, having considered the high risk of graft failure and digestive hemorrhage, MRB surgery was recommended and a 3D reconstruction (Hisense Computer Assisted Surgery System, Hisense, China) from enhanced CT scans was created to facilitate the surgery (Fig. 1). The ethics committee of the hospital approved the study and written informed consent was obtained from the patient.

## 3 Operation

**Figure 2:**
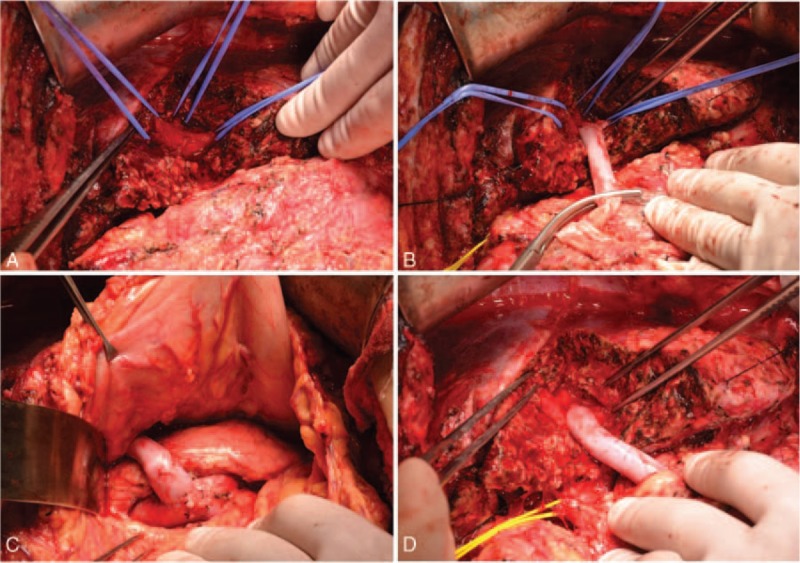
(A) The LPV was buried inside the liver; (B) end-to-side anastomosis of the LPV and the fresh venous allograft; (C) end-to-side anastomosis of the SV and the fresh venous allograft at the inferior margin of the pancreas—the shunt was located behind the pylorus; (D) the state after revascularization with a patent bypass. LPV  =  left portal vein, SV  =  splenic vein.

A Benz incision was made through a previous scar to expose the left side of the liver. Because of severe adhesion and left lobe hyperplasia, Rex's recessus and the LPV were buried deep inside the liver parenchyma. We resected a small portion of the liver segment III and IVB under ultrasound (US) navigation with Cut-Ultrasound Aspiration equipment to find the cystic part of the LPV. Then we separated the SV at the inferior margin of the pancreas. A fresh iliac DCD venous allograft (7 cm long, 1.2 cm in diameter) was procured as the bridge. Before venotomy, we measured the pressure of the SMV at 44 cm H_2_O. End-to-side anastomoses to the LPV (1.5 cm caliber) and the SV (1 cm caliber) were performed by a running suture with 6–0 Prolene nonabsorbable sutures. The graft went through the back of the antral-pyloric region. After revascularization, the SMV pressure dropped to 30 cm H_2_O, which proved the effectiveness of the MRB (Fig. 2).

**Figure 3:**
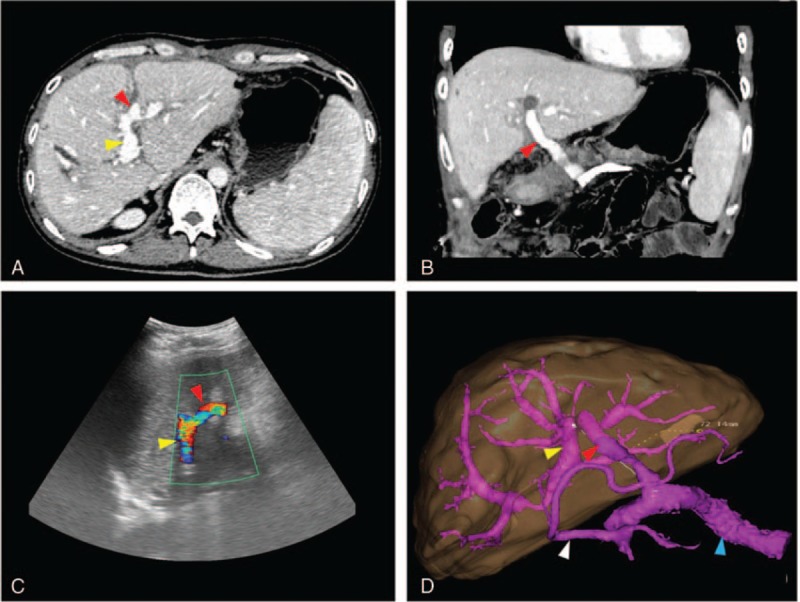
Postoperative enhanced CT scan (A, B) and Doppler US images (C) showed a patent LPV (yellow arrow), MRB (red arrow) and SV (blue arrow); the LPV became dilated and the varix improved (white arrow). On 3D reconstruction, the length of the bypass was shown to be 72.14 mm (D). CT  =  computed tomography, LPV  =  left portal vein, MRB  =  Meso-Rex bypass, SMV  =  superior mesenteric vein, SV  =  splenic vein, 3D  =  three dimensional, US  =  ultrasound.

Postoperative recovery was uneventful. During the first 2 postoperative days, an intravenous heparin infusion (100 units/kg/day) was administered. Then the method of administration was changed to subcutaneous injection of low-molecular-weight heparin (5700 IU, Fraxiparine, GlaxoSmithKline) from the third day onward with an activated partial thromboplastin time target of 50 to 60 seconds. Oral administration of warfarin was initiated 1 week after the operation (International Normalized Ratio of 1.5–2.0) and the anticoagulation therapy lasted for 6 months according to our PVT medication experience. An enhanced CT scan on the seventh day after the operation revealed that the diameter of the LPV had increased to 9.7 mm with improved splanchnic varices. Postoperative 3D reconstruction visually revealed the change from the preoperative state. Two weeks after the patient's operation, blood tests showed: WBC 4.10 × 10^9^/L, PLT 100 × 10^9^/L, NEUT 2.21 × 10^9^/L, ALT 50.3 U/L, AST 33.7 U/L, ALP 274 U/L, GGT 288 U/L, TBIL 10.8 μmol/L, and DBIL 6.4 μmol/L. Doppler US was used regularly to evaluate the patency and blood flow of the shunt. A velocity of 57 cm/s was confirmed 1 month after the operation and the shunt still remained patent at the 6-month postoperative follow-up (Fig. 3).

## 4 Discussion

PVT after liver transplantation is a relatively common but serious complication, which can lead to portal hypertension or a direct loss of the graft. Most patients are asymptomatic during the early stages, but with extended inadequacy of liver perfusion and portal hypertension ascites, variceal bleeding, splenomegaly, and hypersplenism develop. Late-onset PVT occurs less than in early stages and it has been reported that late PVT after a pediatric liver transplant can be extremely well tolerated.^[10]^ However, for adults there may be some differences. In our patient, PVT and cavernous transformation were not revealed during early stage leukopenia and thrombocytopenia by image scanning with moderate liver dysfunction, but it then suddenly presented during follow-up. It taught us a lesson that when signs of hypersplenism occur, patency of the PV should be monitored more rigorously and more frequently, so that the early management of anticoagulation can be timely. Although there was no serious impairment of liver function or a history of bleeding, surgery was chosen because of the high risk of hemorrhage and the susceptible state of a patient with a low white blood cell count on immunosuppressor medication. The signs of sponge-like hepatopetal venous collaterals following PVT at the PV on computed tomography angiography are referred to as cavernous transformation. Interposing a mesenteric-left portal shunt at the level of the umbilical portion of the LPV system (Rex's recessus) was first described by de Ville de Goyet as a solution for the initial cause of portal hypertension.^[11]^ Subsequently, details of this surgical procedure have been adapted for pediatric liver transplantation-induced prehepatic portal hypertension using left jugular vein or venous allografts.^[12]^ This bypass can also be the first choice for portal vascularization in liver transplantation operations as an alternative to truncal PV anastomosis when the donor PV stump has been lost.^[13]^

With a background of left liver hyperplasia, the LPV was deeply embedded in the liver parenchyma and difficult to access. Navigation using Doppler US and preoperative 3D reconstructions were helpful in locating the cystic component during liver dissection.^[14]^ According to historical reports, the Meso-Rex interventions procedure has been demonstrated to be a safe and effective strategy for reconstruction of hepatopetal flow and is not uncommon for pediatric liver transplantation patients; however, as far as we are aware there have been no reports of its use after adult liver transplantation.^[15]^ At present, there have been only reports on 2 cases describing SV-LPV transpositions, but both were combined with splenectomy.^[8,16]^

There is only 1 case report about the interposition of the internal jugular vein from the SV to the LPV without splenectomy (in an 11-year-old boy), in which an intraoperatively detected SMV thrombosis impeded a conventional MRB.^[17]^ For our patient, an aggressive procedure of esophagogastric devascularization was not used because we thought MRB would decompress the PV sufficiently and also the huge spleen volume and complex varices would bear unnecessary risks. We therefore chose a SV–PV bypass, since a patent SV is a good choice as the starting point for a shunt, because there are communicating branches between the SMV and inferior mesenteric vein (IMV), and the IMV converges with the SV. This approach also has the virtue of there being a shorter distance from Rex's recessus to the SV than to the SMV. In our case, we used a fresh iliac DCD venous allograft, but if not available, autologous internal jugular veins can serve as alternative grafts. As result of the intervention, the diameter of the LPV increased to 9.7 mm with improved splanchnic varices and also the hypersplenism improved with elevated WBC and PLT counts, but there was no obvious change in the size of the spleen. A velocity of blood flow of 57 cm/s was confirmed 1 month after the operation and the shunt remained patent at the 6-month postoperative follow-up.

## References

[1] Kuy S Dua A Rieland J Cavernous transformation of the portal vein. J Vasc Surg 2016;63:529. 26804220 10.1016/j.jvs.2014.05.013

[2] Takahashi Y Nishimoto Y Matsuura T Surgical complications after living donor liver transplantation in patients with biliary atresia: a relatively high incidence of portal vein complications. Pediatr Surg Int 2009;25:745–51. 19655151 10.1007/s00383-009-2430-y

[3] De Gaetano AM Lafortune M Patriquin H Cavernous transformation of the portal vein: patterns of intrahepatic and splanchnic collateral circulation detected with Doppler sonography. AJR Am J Roentgenol 1995;165:1151–5. 7572494 10.2214/ajr.165.5.7572494

[4] de Ville de Goyet J Alberti D Clapuyt P Direct bypassing of extrahepatic portal venous obstruction in children: a new technique for combined hepatic portal revascularization and treatment of extrahepatic portal hypertension. J Pediatr Surg 1998;33:597–601. 9574759 10.1016/s0022-3468(98)90324-4

[5] de Ville de Goyet J Alberti D Falchetti D Treatment of extrahepatic portal hypertension in children by mesenteric-to-left portal vein bypass: a new physiological procedure. Eur J Surg 1999;165:777–81. 10494645 10.1080/11024159950189573

[6] Krebs-Schmitt D Briem-Richter A Grabhorn E Effectiveness of Rex shunt in children with portal hypertension following liver transplantation or with primary portal hypertension. Pediatr Transplant 2009;13:540–4. 19210267 10.1111/j.1399-3046.2008.01109.x

[7] Chen W Rodriguez-Davalos MI Facciuto ME Experience with duplex sonographic evaluation of Meso-Rex bypass in extrahepatic portal vein obstruction. J Ultrasound Med 2011;30:403–9. 21357564 10.7863/jum.2011.30.3.403

[8] Chiu B Pillai SB Sandler AD Experience with alternate sources of venous inflow in the Meso-Rex bypass operation: the coronary and splenic veins. J Pediatr Surg 2007;42:1199–202. 17618880 10.1016/j.jpedsurg.2007.02.033

[9] Ha TY Kim KM Ko GY Variant Meso-Rex bypass with transposition of abdominal autogenous vein for the management of idiopathic extrahepatic portal vein obstruction: a retrospective observational study. BMC Surg 2015;15:15116. 10.1186/s12893-015-0101-6 PMC4609139 26475346

[10] Vannevel G Clapuyt P Reding R Spontaneous meso-portal shunt following orthotopic liver transplantation in a child. Pediatr Radiol 2010;40 suppl 1:S92–4. 20596702 10.1007/s00247-010-1758-8

[11] de Ville de Goyet J Clapuyt P Otte JB Extrahilar mesenterico-left portal shunt to relieve extrahepatic portal hypertension after partial liver transplant. Transplantation 1992;53:231–2. 1733076

[12] di Francesco F Grimaldi C de Ville de Goyet J Meso-Rex bypass--a procedure to cure prehepatic portal hypertension: the insight and the inside. J Am Coll Surg 2014;218:e23–36. 24326080 10.1016/j.jamcollsurg.2013.10.024

[13] Rivera J Fusaro F de Magnee C Meso-Rex shunt for immediate portal revascularization in pediatric liver transplantation: first report. Pediatr Transplant 2012;16:E235–7. 21923884 10.1111/j.1399-3046.2011.01576.x

[14] Chen G Yang SZ Wu GQ Development and clinical application of 3D operative planning system of live in virtual reality environments. Zhonghua Wai Ke Za Zhi 2009;47:1620–3. 20137395

[15] de Ville de Goyet J Lo Zupone C Grimaldi C Meso-Rex bypass as an alternative technique for portal vein reconstruction at or after liver transplantation in children: review and perspectives. Pediatr Transplant 2013;17:19–26. 22943796 10.1111/j.1399-3046.2012.01784.x

[16] Chen VT Wei J Liu YC A new procedure for management of extrahepatic portal obstruction. Proximal splenic-left intrahepatic portal shunt. Arch Surg 1992;127:1358–60. 1444799 10.1001/archsurg.1992.01420110106021

[17] Salzedas-Netto AA Duarte AA Linhares MM Variation of the Rex shunt for treating concurrent obstruction of the portal and superior mesenteric veins. J Pediatr Surg 2011;46:2018–20. 22008343 10.1016/j.jpedsurg.2011.07.002

